# Supplementary material for: Discovery and application of insertion-deletion (INDEL) polymorphisms for QTL mapping of early life-history traits in Atlantic salmon
Source: BMC Genomics. 2010 Mar 8;11:156. doi: 10.1186/1471-2164-11-156 (PMC2838853; doi:10.1186/1471-2164-11-156)
Supplement: Additional file 2 — Information on developed 76 locus single-run INDEL panel in Atlantic salmon. Information on fluorescence labeling, primer concentrations, PCR pooling and links to alignments, INDEL motifs and GENESCAN (Burge and Karlin 1997) predictions of genes/exons are available in html format. [file 1471-2164-11-156-S2.ZIP › Additionalfile2/table16424.html]

Contig Alignment

```
Base	A B C D E F   
1	. C . . . .    
2	. C . . . .    
3	. C . . . .    
4	. C . . . .    
5	. C . . . .    
6	. C . . . .    
7	. A . . . .    
8	. A . . . .    
9	. A . . . .    
10	. G . . . .    
11	. T . . . .    
12	. T . . . .    
13	. C . . . .    
14	. C . . . .    
15	. C . . . .    
16	. T . . . .    
17	G G . . . .    
18	G G . . . .    
19	A A . . . .    
20	G G . . . .    
21	G G . . . .    
22	A A . . . .    
23	T T . . . .    
24	C C . . . .    
25	C C . . . .    
26	C C . . . .    
27	A A . . . .    
28	G G . . . .    
29	G G . . . .    
30	A A . . . .    
31	G G . . . .    
32	C C . . . .    
33	T T . . . .    
34	T T . . . .    
35	T T . . . .    
36	T T . . . .    
37	G G . . . .    
38	T T . . . .    
39	C C . . . .    
40	C C . . . .    
41	T T . . . .    
42	C C . . . .    
43	C C . . . .    
44	T T . . . .    
45	T T . . . .    
46	A A . . . .    
47	G G . . . .    
48	A A . . . .    
49	A A . . . .    
50	T T . . . .    
51	A A . . . .    
52	C C . . . .    
53	A A . . . .    
54	C C . . . .    
55	T T . . . .    
56	G G . . . .    
57	T T . . . .    
58	A A . . . .    
59	G G . . . .    
60	A A . . . .    
61	A A . . . .    
62	T T . . . .    
63	A A . . . .    
64	T T . . . .    
65	A A . . . .    
66	T T . . . .    
67	T T . . . .    
68	- N . . . .    
69	T T . . . .    
70	G G . . . .    
71	A A . . . .    
72	A A . . . .    
73	A A . . . .    
74	A A . . . .    
75	G G . . . .    
76	A A . . . .    
77	T T . . . .    
78	G G . . . .    
79	C C . . . .    
80	A A . . . .    
81	A A . . . .    
82	G G . . . .    
83	A A . . . .    
84	G G . . . .    
85	G G . . . .    
86	A A . . . .    
87	G G . . . .    
88	A A . . . .    
89	G G . . . .    
90	A A . . . .    
91	G G . . . .    
92	A A . . . .    
93	G G . . . .    
94	C C . . . .    
95	T T . . . .    
96	G G . . . .    
97	A A . . . .    
98	G G . . . .    
99	T T . . . .    
100	T T . . . .    
101	C C . . . .    
102	A A . . . .    
103	C C . . . .    
104	C C . . . .    
105	T T . . . .    
106	G G . . . .    
107	T T . . . .    
108	G G . . . .    
109	A A . . . .    
110	T T . . . .    
111	G G . . . .    
112	G G . . . .    
113	A A . . . .    
114	G G . . . .    
115	G G . . . .    
116	T T . . . .    
117	C C . . . .    
118	G G . . . .    
119	T T . . . .    
120	G G . . . .    
121	T T . . . .    
122	G G . . . .    
123	A A . . . .    
124	T T . . . .    
125	G G . . . .    
126	A A . . . .    
127	G G . . . .    
128	G G . . . .    
129	G G . . . .    
130	C C . . . .    
131	G G . . . .    
132	A A . . . .    
133	C C . . . .    
134	A A . . . .    
135	G G . . . .    
136	G G . . . .    
137	G G . . . .    
138	A A . . . .    
139	T T . . . .    
140	A A . . . .    
141	G G . . . .    
142	C C . . . .    
143	C C . . . .    
144	C C . . . .    
145	T T . . . .    
146	A A . . . .    
147	C C . . . .    
148	G G . . . .    
149	G G . . . .    
150	A A . . . .    
151	C C . . . .    
152	G G . . . .    
153	A A . . . .    
154	G G . . . .    
155	G G . . . .    
156	C C . . . .    
157	T T . . . .    
158	A A A . A .    
159	G G G . G .    
160	G G G . G .    
161	G G G . G .    
162	G G G . G .    
163	G G G . G .    
164	A A A . A .    
165	C C C . C .    
166	A A A . A .    
167	C C C . C .    
168	T T T . T .    
169	G G G . G .    
170	G G G . G .    
171	A A A . A .    
172	G G G . G .    
173	T T T . T .    
174	G G G . G .    
175	A A A . A .    
176	A A A . A .    
177	A A A . A .    
178	A A A . A .    
179	T T T . T .    
180	A A A . A .    
181	T T T . T .    
182	A A A . A .    
183	A A A . A .    
184	C C C . C .    
185	T T T . T .    
186	G G G . G .    
187	A A A . A .    
188	T T T . T .    
189	C C C . C C    
190	A A A . A A    
191	A A A . A A    
192	A A A . A A    
193	T T T . T T    
194	A A A . A A    
195	A A A . A A    
196	G G G . G G    
197	A A A . A A    
198	C C C . C C    
199	G G G . G G    
200	- - T . T T  2	 indel  
201	- - G . G G  2	 indel  
202	T T T . T T    
203	T T T . T T    
204	T T T . T T    
205	T T T . T T    
206	A A A . A A    
207	T T T . T T    
208	G G G . G G    
209	G G G . G G    
210	A A A . A A    
211	G G G . A G    
212	A A A . A A    
213	A A A . A A    
214	T T T . T T    
215	C C C . C C    
216	T T T . T T    
217	G G G . G G    
218	T T T . T T    
219	G G G . G G    
220	G G G . G G    
221	T T T . T T    
222	T T T . T T    
223	G G G . G G    
224	A A A . A A    
225	A A A . A A    
226	G G G . G G    
227	C C C . C C    
228	T T T . T T    
229	T T T . T T    
230	T T T . T T    
231	T T T . T T    
232	T T T . T T    
233	C C C . C C    
234	T T T . T T    
235	C C C . C C    
236	G G G . G G    
237	C C C . C C    
238	A A A . A A    
239	C C C . C C    
240	T T T T T T    
241	A A A A A A    
242	A A A A A A    
243	A A A A A A    
244	A A A A A A    
245	A A A A A A    
246	T T T T T T    
247	T T T T T T    
248	C C C C C C    
249	A A A A A A    
250	C C C C C C    
251	A A A A A A    
252	T T T T T T    
253	T T T T T T    
254	G G G G G G    
255	A A A A A A    
256	T T T T T T    
257	A A A A A A    
258	T T T T T T    
259	G G G G G G    
260	G G G G G G    
261	A A A A A A    
262	G G G G G G    
263	A A A A A A    
264	T T T T T T    
265	G G G G G G    
266	T T T T T T    
267	G G G G G G    
268	T T T T T T    
269	C C C C C C    
270	A A A A A A    
271	A A A A A A    
272	T T T T T T    
273	A A A A A A    
274	G G G G G G    
275	A A A A A A    
276	T T T T T T    
277	G G G G G G    
278	T T T T T T    
279	C C C C C C    
280	A A A A A A    
281	A A A A A A    
282	T T T T T T    
283	G G G G G G    
284	C C C C C C    
285	G G G G G G    
286	C C C C C C    
287	A A A A A A    
288	T T T T T T    
289	C C C C C C    
290	A A A A A A    
291	T T T T T T    
292	G G G G G G    
293	T T T T T T    
294	T T T T T T    
295	G G G G G G    
296	G G G G G G    
297	T T T T T T    
298	G G G G G G    
299	A A A A A A    
300	C C C C C C    
301	C C C C C C    
302	T T T T T T    
303	A A A A A A    
304	G G G G G G    
305	A A A A A A    
306	A A A A A A    
307	A A A A A A    
308	G G G G G G    
309	T T T T T T    
310	T T T T T T    
311	C C C C C C    
312	A A A A A A    
313	A A A A A A    
314	T T T T T T    
315	A A A A A A    
316	T T T T T T    
317	G G G G G G    
318	T T T T T T    
319	T T T T T T    
320	C C C C C C    
321	C C C C C C    
322	A A A A A A    
323	G G G G G G    
324	T T T T T T    
325	A A A A A A    
326	A A A A A A    
327	T T T T T T    
328	T T T T T T    
329	C C C C C C    
330	A A A A A A    
331	C C C C C C    
332	A A A A A A    
333	G G G G G G    
334	T T T T T T    
335	A A A A A A    
336	C C C C C C    
337	C C C C C C    
338	A A A A A A    
339	G G G G G G    
340	T T T T T T    
341	C C C C C C    
342	A A A A A A    
343	T T T T T T    
344	G G G G G G    
345	A A A A A A    
346	A A A A A A    
347	A A A A A A    
348	G G G G G G    
349	G G G G G G    
350	A A A A A A    
351	T T T T T T    
352	A A A A A A    
353	T T T T T T    
354	G G G G G G    
355	A A A A A A    
356	T T T T T T    
357	T T T T T T    
358	G G G G G G    
359	A A A A A A    
360	A A A A A A    
361	G G G G G G    
362	T T T T T T    
363	G G G G G G    
364	C C C C C C    
365	C C C C C C    
366	A A A A A A    
367	G G G G G G    
368	T T T T T T    
369	T T T T T T    
370	G G G G G G    
371	T T T T T T    
372	G G G G G G    
373	T T T T T T    
374	G G G G G G    
375	A A A A A A    
376	A A A A A A    
377	G G G G G G    
378	T T T T T T    
379	T T T T T T    
380	G G G G G G    
381	T T T T T T    
382	G T T T T T    
383	. T T T T T    
384	. A A A A A    
385	. T T T T T    
386	. G G G G G    
387	. G G G G G    
388	. T T T T T    
389	. A A A A A    
390	. A A A A A    
391	. T T T T T    
392	. C C C C C    
393	. A A A A A    
394	. T T T T T    
395	. A A A A A    
396	. G G G G G    
397	. C C C C C    
398	. A A A A A    
399	. T T T T T    
400	. A A A A A    
401	. G G G G G    
402	. A A A A A    
403	. T T T T T    
404	. A A A A A    
405	. C C C C C    
406	. A A A A A    
407	. G G G G G    
408	. G G G G G    
409	. A A A A A    
410	. A A A A A    
411	. T T T T T    
412	. A A A A A    
413	. G G G G G    
414	. A A A A A    
415	. A A A A A    
416	. A A A A A    
417	. C C C C C    
418	. A A A A A    
419	. T T T T T    
420	. A A A A A    
421	. A A A A A    
422	. A A A A A    
423	. T T T T T    
424	. T T T T T    
425	. A A A A A    
426	. A A A A A    
427	. A A A A A    
428	. C C C C C    
429	. A A A A A    
430	. T T T T T    
431	. G G G G G    
432	. G G G G G    
433	. T T T T T    
434	. C C C C C    
435	. T T T T T    
436	. T T T T T    
437	. A A A A A    
438	. C C C C C    
439	. T T T T T    
440	. T T T T T    
441	. G G G G G    
442	. T T T T T    
443	. C C C C C    
444	. A A A A A    
445	. A A A A A    
446	. C C C C C    
447	. T T T T T    
448	. G G G G G    
449	. G G G G G    
450	. G G G G G    
451	. A A A A A    
452	. C C C C C    
453	. G G G A G    
454	. T T T T T    
455	. C C C C C    
456	. G G G G G    
457	. A A A A A    
458	. T T T T T    
459	. A A A A A    
460	. G G G G G    
461	. C C C C C    
462	. A A A A A    
463	. A A A A A    
464	. G G G G G    
465	. A A A A A    
466	. C C C C C    
467	. A A A A A    
468	. T T T T T    
469	. T T T T T    
470	. T T T T T    
471	. A A A A A    
472	. G G G G G    
473	. T T T T T    
474	. G G G G G    
475	. C C C C C    
476	. T T T T T    
477	. G G G G G    
478	. C C C C C    
479	. G G G G G    
480	. G G G G G    
481	. T T T T T    
482	. T T T T T    
483	. A A A A A    
484	. T T T T T    
485	. G G G G G    
486	. T T T T T    
487	. G G G G G    
488	. T T T T T    
489	. A A A A A    
490	. T T T T T    
491	. T T T T T    
492	. A A A A A    
493	. C C C C C    
494	. G G G G G    
495	. T T T T T    
496	. A A A A A    
497	. G G G G G    
498	. G G G G G    
499	. T T T T T    
500	. G G G G G    
501	. A A A A A    
502	. G G G G G    
503	. G G G G G    
504	. A A A A A    
505	. A A A A A    
506	. A A A A A    
507	. A A A A A    
508	. T T T T T    
509	. T T T T T    
510	. G G G G G    
511	. T T T T T    
512	. A A A A A    
513	. T T T T T    
514	. G G G G G    
515	. T T T T T    
516	. T T T T T    
517	. C C C C C    
518	. T T T T T    
519	. G G G G G    
520	. T T T T T    
521	. G G G G G    
522	. T T T T T    
523	. T T T T T    
524	. C C C C C    
525	. A A A A A    
526	. G G G G G    
527	. C C C C C    
528	. C C C C C    
529	. C C C C C    
530	. A A A A A    
531	. A A A A A    
532	. A A A A A    
533	. A A A A A    
534	. T T T T T    
535	. G G G G G    
536	. T T T T T    
537	. A A A A A    
538	. A A A A A    
539	. T T T T T    
540	. T T T T T    
541	. T T T T T    
542	. T T T T T    
543	. G G G G G    
544	. T T T T T    
545	. T T T T T    
546	. T T T T T    
547	. G G G G G    
548	. C C C C C    
549	. T T T T T    
550	. A A A A A    
551	. G G G G G    
552	. C C C C C    
553	. T T T T T    
554	. A A A A A    
555	. C C C C C    
556	. C C C C C    
557	. A A A A A    
558	. G G G G G    
559	. T T T T T    
560	. T T T T T    
561	. T T T T T    
562	. T T T T T    
563	. G G G G G    
564	. C C C C C    
565	. T T T T T    
566	. G G G G G    
567	. C C C C C    
568	. C C C C C    
569	. T T T T T    
570	. T T T T T    
571	. T T T T T    
572	. T T T T T    
573	. C C C C C    
574	. C C C C C    
575	. G G G G G    
576	. G G G G G    
577	. A A A A A    
578	. T T T T T    
579	. C C C C C    
580	. T T T T T    
581	. G G G G G    
582	. C C C C C    
583	. T T T T T    
584	. G G G G G    
585	. T T T T T    
586	. C C C C C    
587	. A A A A A    
588	. T T T T T    
589	. T T T T T    
590	. T T T T T    
591	. C C C C C    
592	. T T T T T    
593	. C C C C C    
594	. A A A A A    
595	. T T T T T    
596	. T T T T T    
597	. C C C C C    
598	. T T T T T    
599	. G G G G G    
600	. A A A A A    
601	. C C C C C    
602	. T T T T T    
603	. G G G G G    
604	. G G G G G    
605	. T T T T T    
606	. A A A A A    
607	. T T T T T    
608	. G G G G G    
609	. T T T T T    
610	. A A A A A    
611	. A A A A A    
612	. T T T T T    
613	. G G G G G    
614	. A A A A A    
615	. G G G G G    
616	. C C C C C    
617	. A A A A A    
618	. G G G G G    
619	. G G G G G    
620	. G G G G G    
621	. T T T T T    
622	. A A A A A    
623	. A A A A A    
624	. A A A A A    
625	. T T T T T    
626	. A A A A A    
627	. T T T T T    
628	. T T T T T    
629	. C C C C C    
630	. A A A A A    
631	. A A A A A    
632	. A A A A A    
633	. T T T T T    
634	. A A A A A    
635	. T T T T T    
636	. A A A A A    
637	. A A A A A    
638	. C C C C C    
639	. A A A A A    
640	. T T T T T    
641	. A A A A A    
642	. A A A A A    
643	. G G G G G    
644	. T T T T T    
645	. T T T T T    
646	. A A A A A    
647	. A A A A A    
648	. T T T T T    
649	. C C C C C    
650	. A A A A A    
651	. T T T T T    
652	. G G G G G    
653	. . T T T T    
654	. . T T T T    
655	. . T T T T    
656	. . T T T T    
657	. . C C C C    
658	. . A A A A    
659	. . T T T T    
660	. . G G G G    
661	. . T T T T    
662	. . T T T T    
663	. . T T T T    
664	. . C C C C    
665	. . T T T T    
666	. . G G G G    
667	. . G G G G    
668	. . C C C C    
669	. . T T T T    
670	. . T T T T    
671	. . T T T T    
672	. . T T T T    
673	. . A A A A    
674	. . G G G G    
675	. . A A A A    
676	. . G G G G    
677	. . T T T T    
678	. . C C C C    
679	. . T T T T    
680	. . T T T T    
681	. . T T T T    
682	. . A A A A    
683	. . T T T T    
684	. . G G G G    
685	. . T T T T    
686	. . G G G G    
687	. . T T T T    
688	. . A A A A    
689	. . T T T T    
690	. . G G G G    
691	. . T T T T    
692	. . A A A A    
693	. . C C C C    
694	. . A A A A    
695	. . T T T T    
696	. . T T T T    
697	. . G G G G    
698	. . T T T T    
699	. . G G G G    
700	. . T T T T    
701	. . G G G G    
702	. . A A A A    
703	. . A A A A    
704	. . G G G G    
705	. . C C C C    
706	. . A A A A    
707	. . G G G G    
708	. . T T T T    
709	. . C C C C    
710	. . T T T T    
711	. . A A A A    
712	. . T T T T    
713	. . A A A A    
714	. . G G G G    
715	. . G G G G    
716	. . G G G G    
717	. . C C C C    
718	. . T T T T    
719	. . A A A A    
720	. . C C C C    
721	. . A A A A    
722	. . G G G G    
723	. . T T T T    
724	. . G G G G    
725	. . G G G G    
726	. . A A A A    
727	. . T T T T    
728	. . G G G G    
729	. . T T T T    
730	. . T T T T    
731	. . A A A A    
732	. . A A A A    
733	. . A A A A    
734	. . G G G G    
735	. . C C C C    
736	. . C C C C    
737	. . C C C C    
738	. . C C C C    
739	. . C C C C    
740	. . A A A A    
741	. . T T T T    
742	. . G G G G    
743	. . C C C C    
744	. . A A A A    
745	. . G G G G    
746	. . T T T T    
747	. . C C C C    
748	. . T T T T    
749	. . T T T T    
750	. . T T T T    
751	. . T T T T    
752	. . T T T T    
753	. . A A A A    
754	. . A A A A    
755	. . T T T T    
756	. . T T T T    
757	. . G G G G    
758	. . T T T T    
759	. . A A A A    
760	. . T T T T    
761	. . T T T T    
762	. . T T T T    
763	. . T T T T    
764	. . T T T T    
765	. . A A A A    
766	. . A A A A    
767	. . A A A A    
768	. . A A A A    
769	. . T T T T    
770	. . C C C C    
771	. . A A A A    
772	. . T T T T    
773	. . C C C C    
774	. . A A A A    
775	. . C C C C    
776	. . T T T T    
777	. . G G G G    
778	. . T T T T    
779	. . A A A A    
780	. . T T T T    
781	. . G G G G    
782	. . C C C C    
783	. . C C C C    
784	. . T T T T    
785	. . A A A A    
786	. . A A A A    
787	. . T T T T    
788	. . T T T T    
789	. . C C C C    
790	. . A A A A    
791	. . G G G G    
792	. . T T T T    
793	. . A A A A    
794	. . A A A A    
795	. . A A A A    
796	. . T T T T    
797	. . C C C C    
798	. . A . A A    
799	. . C . C C    
800	. . T . T T    
801	. . C . C C    
802	. . T . T T    
803	. . G . G G    
804	. . T . T T    
805	. . G . G G    
806	. . T . T T    
807	. . T . T T    
808	. . A . A A    
809	. . A . A A    
810	. . T . T T    
811	. . T . T T    
812	. . T . T T    
813	. . C . C C    
814	. . A . . A    
815	. . T . . T    
816	. . G . . G    
817	. . A . . A    
818	. . A . . A    
819	. . A . . A    
820	. . A . . A    
821	. . T . . T    
822	. . A . . A    
823	. . A . . A    
824	. . T . . T    
825	. . A . . A    
826	. . C . . C    
827	. . A . . A    
828	. . T . . T    
829	. . T . . T    
830	. . T . . T    
831	. . A . . A    
832	. . T . . T    
833	. . A . . A    
834	. . A . . A    
835	. . T . . T    
836	. . A . . A    
837	. . A . . A    
838	. . T . . T    
839	. . T . . T    
840	. . T . . T
```
